# Juvenile Nasopharyngeal Angiofibroma: A Case Report of a Rare Adolescent Head and Neck Tumor

**DOI:** 10.7759/cureus.33633

**Published:** 2023-01-11

**Authors:** Sai Santhosha Mrudula Alla, Deekshitha Alla, Vysakh Ratheesh, Pavan Kumar Ramineni, Ajay Singh, Vaishnavi K, Ayesha Siddiqua

**Affiliations:** 1 Department of Medicine, Andhra Medical College, Visakhapatnam, IND; 2 Internal Medicine, Medical University - Pleven, Pleven, BGR; 3 Internal Medicine, A. C. Subba Reddy Government Medical College, Nellore, IND; 4 Internal Medicine, Shri Ram Murti Smarak Institute of Medical Sciences, Bareilly, IND; 5 Internal Medicine, Sapthagiri Institute of Medical Science and Research Centre, Bengaluru, IND; 6 Department of Medicine, Shadan Institute of Medical Sciences, Hyderabad, IND

**Keywords:** surgical resection, androgens, flutamide, computed tomography, juvenile nasopharyngeal angiofibroma

## Abstract

Juvenile nasopharyngeal angiofibroma (JNA) is a rare benign head and neck tumor. We report a rare case of JNA, provide a brief literature review, and treatment options, and emphasized the role of flutamide as pre-surgical medication for tumor regression. JNA primarily affects adolescent males aged 14 to 25 years. There are various theories explaining the formation of the tumor. However, sex hormones are found to play a crucial role in the etiology of the tumor. In recent years testosterone and dihydrotestosterone receptors have been identified on the tumor thus suggesting the strong influence of hormones. This permits the use of flutamide, an androgen receptor blocker, as adjuvant therapy for the treatment of JNA. This is a case of a 12-year-old boy who presented to the hospital with right-sided nasal obstruction, epistaxis, watery nasal discharge, and a mass in the right nasal cavity for two months. Diagnostic nasal endoscopy, ultrasonography, computed tomography, and magnetic resonance imaging were done. These investigations confirmed the diagnosis of JNA stage IV. The patient was started on treatment with flutamide for tumor regression.

## Introduction

Epistaxis or bleeding from the nose is a common emergency that presents to primary care clinics, responsible for one in every 200 visits to the emergency department. Various factors that may precipitate epistaxis are trauma, metabolic disorders, autoimmune conditions, vascular abnormalities, neoplasia, and inflammatory and congenital disorders. Though traumatic causes like nose pricking are primarily responsible for epistaxis in children, it shouldn’t be undermined as it can be due to several underlying systemic disorders. Juvenile nasopharyngeal angiofibroma (JNA) is a rare benign head and neck tumor that presents with epistaxis. It represents 0.05% and 0.5% of head and neck tumors and the incidence is between 1:6000 and 1:55000 with almost exclusive occurrence in male adolescents [1]. Incidence is slightly higher in Egypt and India compared to that in the USA and Europe [2].

We present a case of a 12-year-old boy who had an extraoral facial swelling diagnosed as JNA stage IV. We aim to focus on the literature review and widen the gaze of clinicians on this rare entity. We also emphasize the role of flutamide as a pre-surgical medication for tumor shrinkage.

## Case presentation

**Figure 1:**
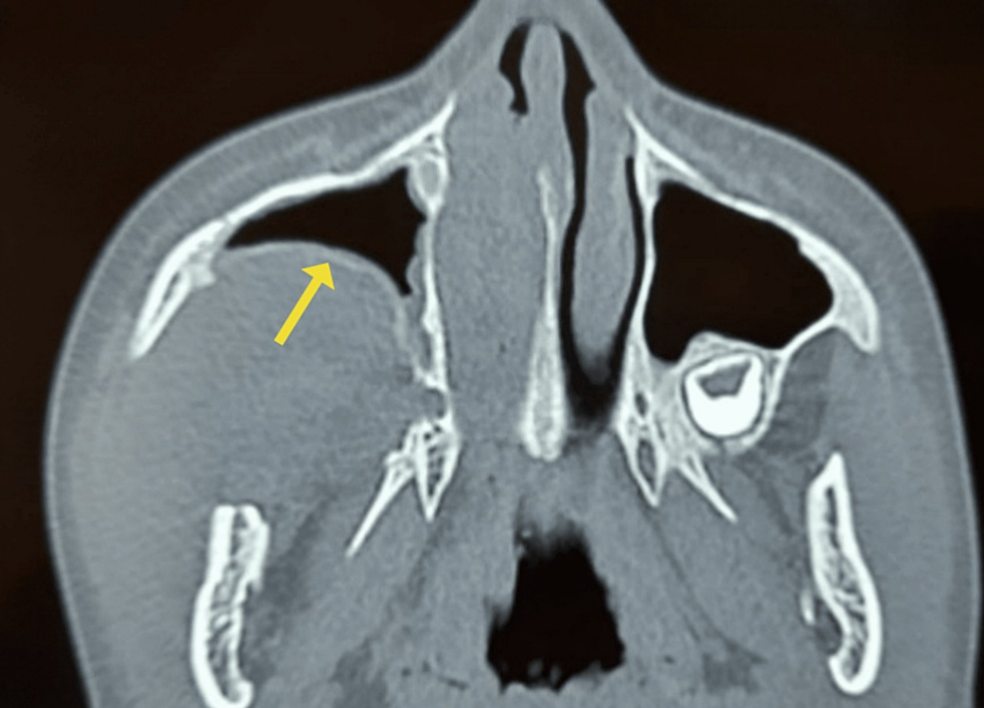
Well-defined soft density mass lesion causing anterior bowing of posterior wall of the maxillary sinus as seen in computed tomography (axial view)

**Figure 2:**
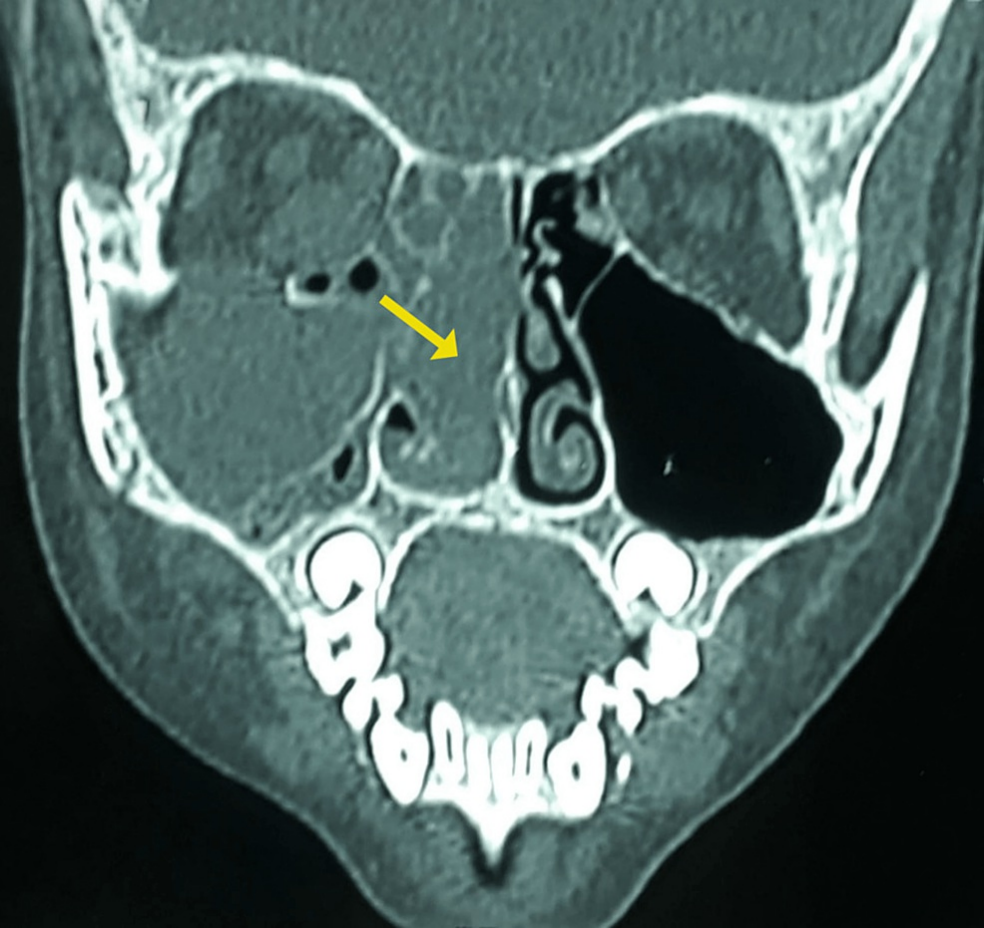
Coronal view showing soft density mass lesion occupying right nasal cavity and maxillary sinus as seen in computed tomography of paranasal sinuses

**Figure 3:**
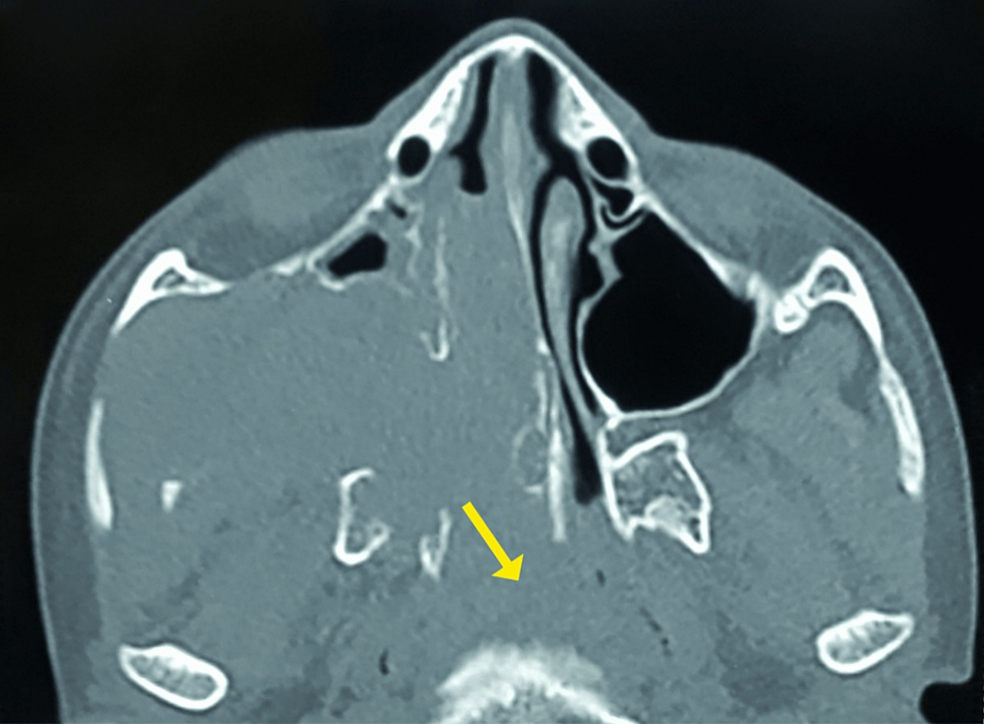
Coronal view showing soft density mass lesion occupying nasopharynx as seen in computed tomography of paranasal sinuses

**Figure 4:**
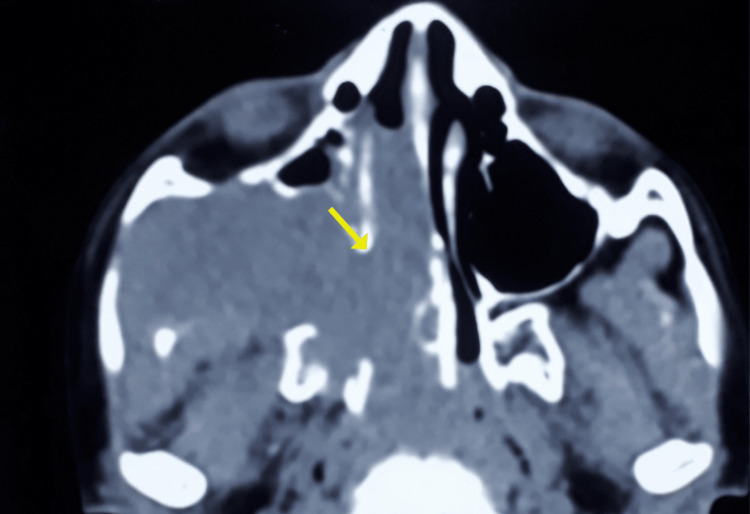
Contrast-enhanced computed tomography of paranasal sinuses showing bony resorption

**Figure 5:**
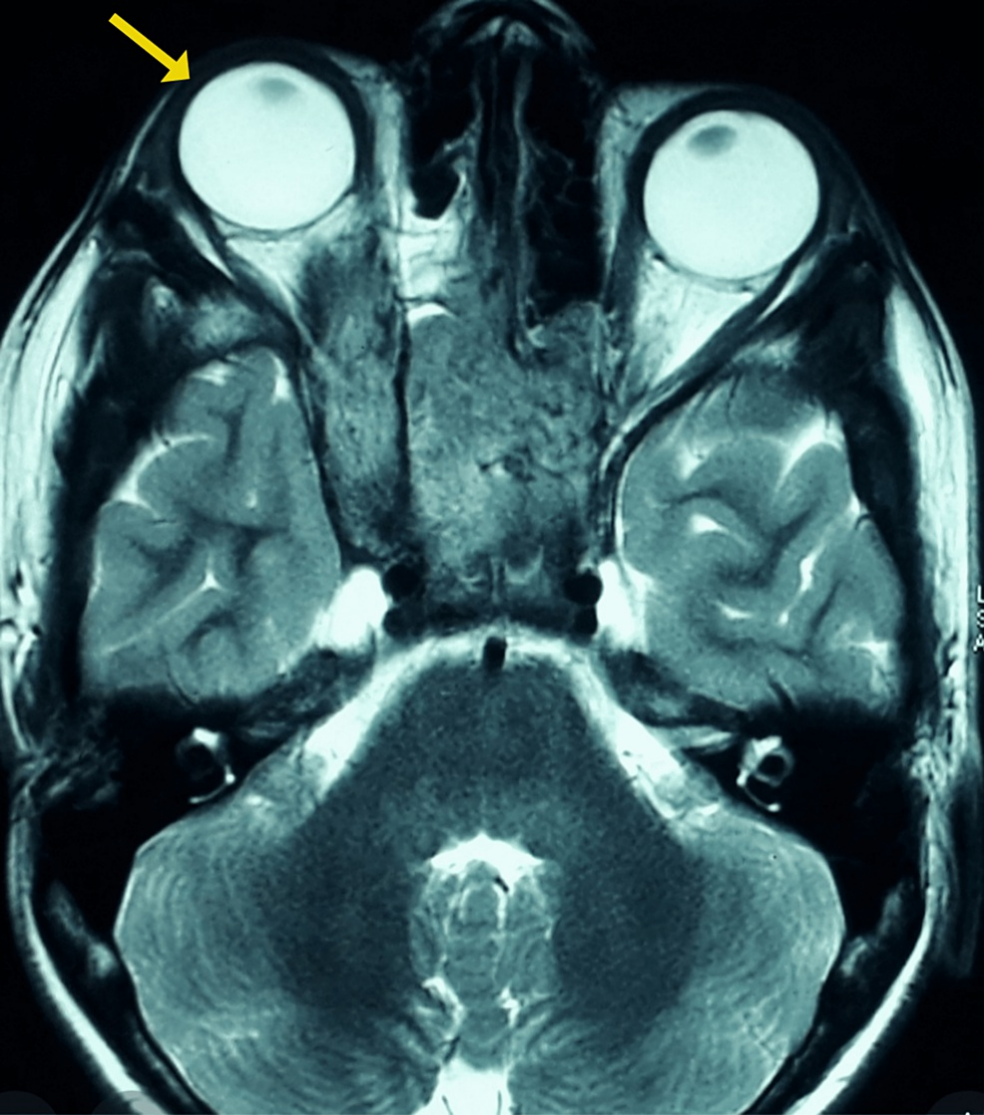
T2 weighted image showing an iso-hypointense mass lesion causing right globe proptosis in magnetic resonance imaging of the brain, paranasal sinuses, and orbits

A 12-year-old boy was referred to us from a local private clinic for swelling on the right side of his face. The child presented with complaints of right-sided nasal obstruction, epistaxis, watery nasal discharge for two months, and a mass in the right nasal cavity. He frequently experienced painless epistaxis episodes that lasted 15 to 20 minutes each, resulting in blood loss of 30 to 40 ml. There was clear mouth breathing, especially during sleep. The patient did not express any headache complaints. History reveals no similar episodes of epistaxis two months ago. Upon extra oral examination, there was gross asymmetry of the face due to swelling over the right cheek. The swelling extended from the infraorbital margin superiorly to the lower border of the mandible inferiorly, from the ala of the nose and corner of the mouth anteriorly to the tragus of the ear and ramus of the mandible posteriorly. It was 7 cm × 6 cm in size, smooth with well-defined margins, and firm in consistency. It was tender, compressible, and pulsatile on palpation. Intraoral examination was normal. The patient showed no pallor, and the results of the routine hematological tests were within normal limits. Diagnostic nasal endoscopy revealed a congested right nasal cavity with the presence of a compressible mass that bled on touch. Fine needle aspiration revealed only blood. Ultrasound neck revealed a well-defined hypoechoic lesion of size 35 × 20 mm with internal vascularity in deep tissues of the cheek on the right side with proximity to the right maxilla. Computed tomography of paranasal sinuses showed a well-defined non-encapsulated soft tissue density mass lesion of size 69.5 ×56 × 30 mm epi centered in the right sphenopalatine foramen extending laterally into the pterygomaxillary fascia, infratemporal fossa causing anterior bowing of maxillary sinus posterior wall as seen in Figure 1 and Figure 2. Medially, it showed extension into the nasopharynx and posterior ethmoid air cells as seen in Figure 3. There is bony remodeling with areas of resorption without any destruction as seen in Figure 4. There is right globe proptosis and further extension of the tumor into the right cavernous sinus in the extradural space of the right temporal lobe as seen in Figure 5. These features are highly suggestive of JNA stage IV. Magnetic resonance imaging of the brain, paranasal sinuses, and orbits showed a heterogeneously hypo intense mass lesion in T1 weighted images and iso-hypointense mass lesion in T2 weighted images involving the sphenoid sinuses and posterior ethmoidal sinuses bilaterally and extending into the posterior nasal cavity on the right side.

**Figure 6:**
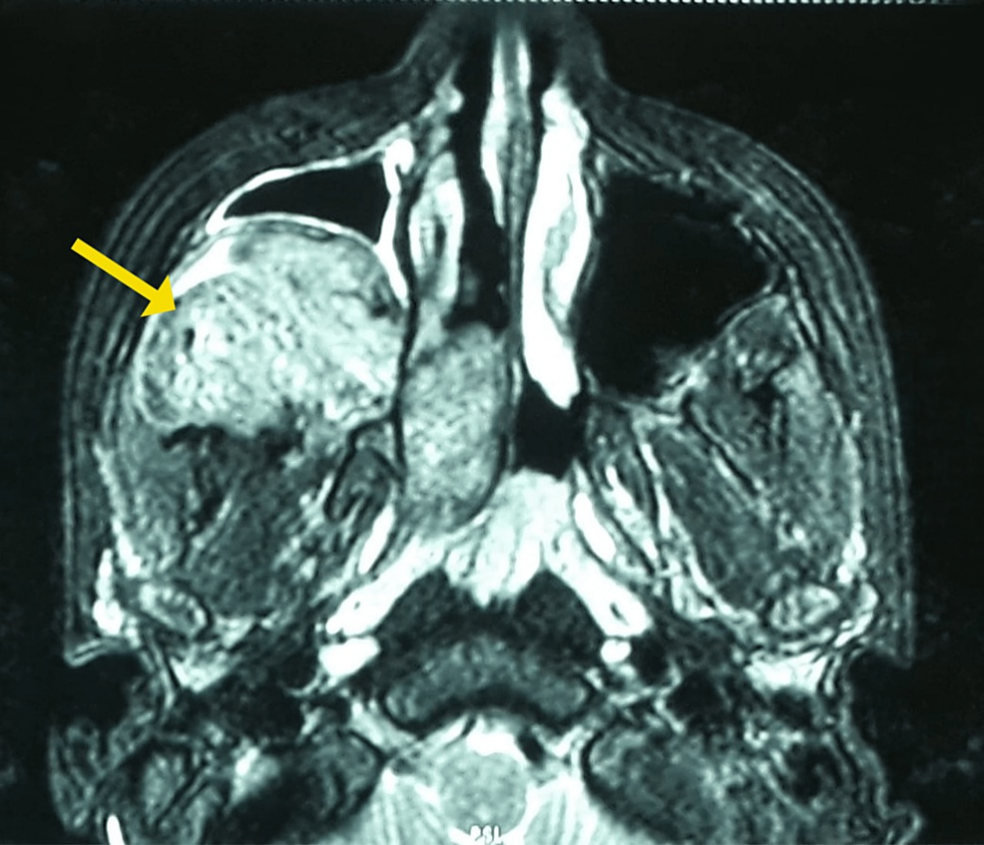
T1 weighted image showing a heterogeneously hypointense mass lesion occupying the right maxillary sinus in magnetic resonance imaging of paranasal sinuses

**Figure 7:**
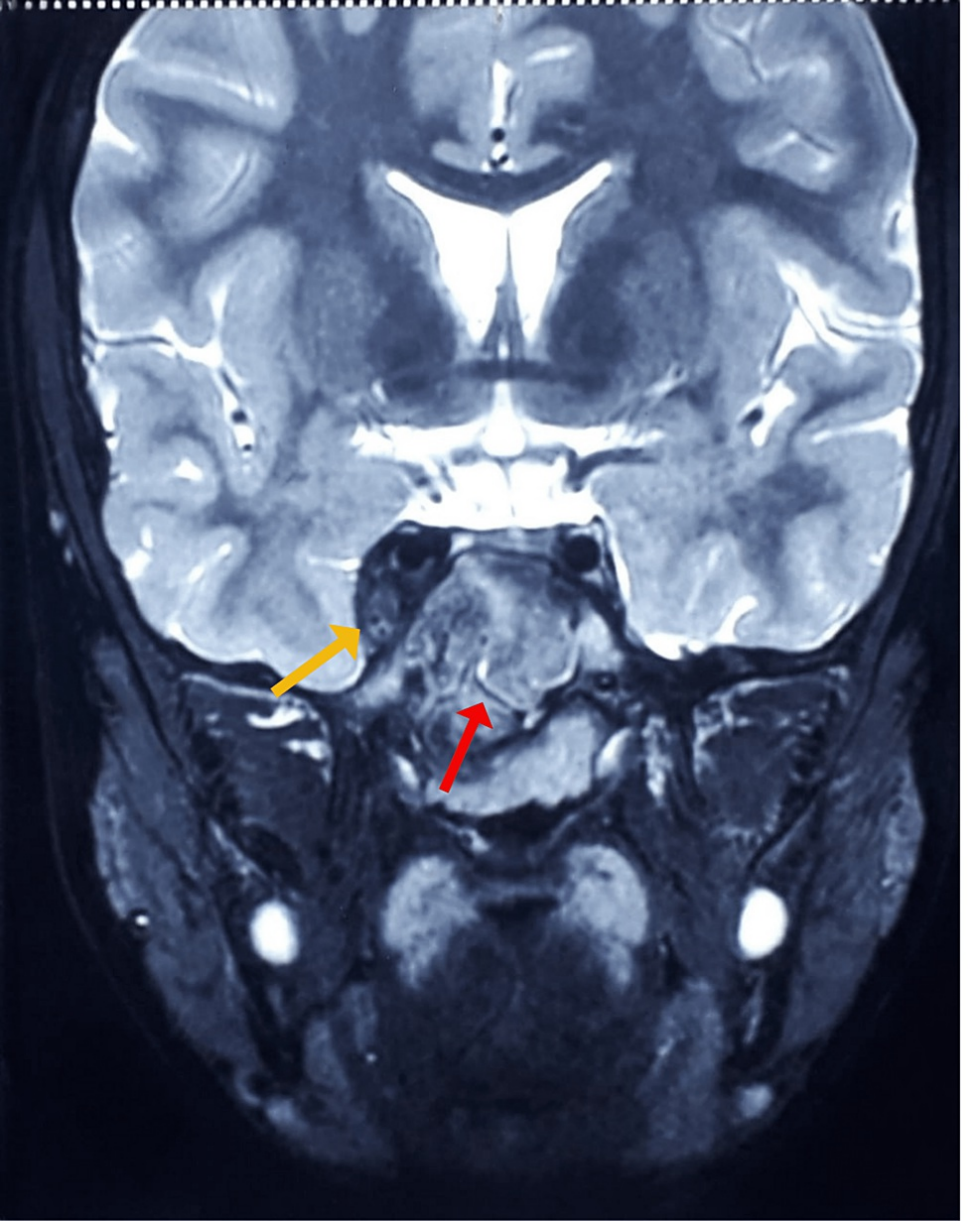
T2 weighted image showing an iso-hypointense mass lesion involving sphenoid sinuses (red arrow) and invading right cavernous sinus (yellow arrow) in magnetic resonance imaging of the brain, paranasal sinuses, and orbits

Figure 6 and Figure 7 show the mass extended into the right superior orbital fissure antero-superiorly. Inferno-laterally, it extended into the pterygopalatine fossa, retro maxillary pad of fat, buccal space, and entire masticator space projecting medially into para pharyngeal space and nasopharyngeal cavity. The lesion shows homogenous enhancement in the post-contrast sequences. The patient was kept under treatment with flutamide for the regression of the tumor to facilitate surgical excision and decrease surgical morbidity. The patient was lost to follow-up at the hospital.

## Discussion

JNA is a relatively uncommon benign tumor that chiefly affects adolescent boys aged 14 to 25 years. The tumor originates near the sphenopalatine foramen and extends toward the surrounding structures [3]. It appears as a spongy, polypoidal mass that ranges in color from red to grey and is not encapsulated. It is typically supplied by the external carotid vasculature and occasionally by the internal carotid artery branches. The tumor consists of fibrous stroma and is densely vascular accounting for profuse bleeding. JNA is usually associated with insertions and deletions in the regions of 4q, 6q, 8q, 12, 17, 22q, and X and Y. There are various theories regarding the formation of angiofibroma. According to a study, vascular endothelial growth factor (VEGF) and platelet-derived growth factor (PDGF) have been found to play a crucial role in the neo-angiogenesis of the tumor. The tumor also showed an increased number of receptors for VEGF when compared to the normal nasal mucosa [4]. Another study by Martin et al. presented the relationship between sex hormones and the etiology of the tumor. It was observed in the study that the tumor was limited to young males and it regressed only after secondary sex characteristics developed despite the radiation therapy. It regressed more readily with x-ray therapy when puberty was hastened by the administration of androgens [5]. In recent years, researchers have developed a better understanding of the receptors of sex hormones found on the surface of the tumor. Testosterone and dihydrotestosterone receptors have been identified on the tumor thus suggesting the strong influence of hormones, especially androgens on the tumor. This permits the use of flutamide, an androgen receptor blocker, as adjuvant therapy for the treatment of JNA. Flutamide is a non-steroidal anti-androgen drug that competitively binds androgen receptors throughout the body. It is a pure anti-androgen drug without agonistic effects which inhibits the negative feedback of testosterone on gonadotropin secretion, resulting in a rise in testosterone and gonadotropins. Several studies have demonstrated the role of flutamide as a pre-operative medication. Gates et al. found a mean tumor shrinkage of 44% in four cases after pre-surgical treatment with flutamide [6]. Another study by Thakar et al. found that pre-pubertal cases did not show a significant response to flutamide suggesting that high testosterone levels are required for the response to flutamide [7]. The treatment options available are surgical resection, radiotherapy, chemotherapy, and hormone therapy. Recent treatment modality involves visualization of feeding vessels through digital subtraction angiography and embolization of vessels before surgery. Surgical resection of the tumor is the most common method followed. Preoperatively, imaging is done to accurately assess the size, location, and spread of the tumor [8]. The tumor can be resected using the endonasal-endoscopic approach also. Radiotherapy can be used in cases where the residual or recurrent lesions spread extensively into the skull base affecting neurovascular structures and thus cannot be surgically treated. The surgical approach allows the effective closure of any skull base defects. However, complications like osteomyelitis, cerebrospinal fluid (CSF) leak, soft tissue defects, soft palate perforations, keratopathy, and amaurosis can occur. The prognosis of nasopharyngeal angiofibroma depends on the size and location of the tumor. Most tumors can be effectively cured with surgical resection or radiotherapy.

## Conclusions

JNA is a locally aggressive, slowly growing tumor with a high propensity to persistence and recurrence. Imaging is the sole method that can identify the lesion early, and it also plays a key role in deciding on surgical procedures and determining the prognosis. Flutamide is effective in the post-pubertal population and is used pre-operatively for the regression of the tumor. However, surgical excision is the main treatment modality that depends on tumor extension.
